# Editorial

**Published:** 2012-02-24

**Authors:** Usha Mohan Das

## Abstract

Management of the grossly carious primary molar is a common but sometimes challenging aspect of dental care for young children. Regrettably, the caries experience of 5 to 6 year olds looks unlikely to improve in the foreseeable future. It is, therefore, essential that clinicians are both content and competent in selecting and undertaking the most appropriate treatment for grossly carious primary molars.

## Sealing in Caries in Primary Molars? Healing redefined?

Management of the grossly carious primary molar is a common but sometimes challenging aspect of dental care for young children. Regrettably, the caries experience of 5 to 6 year olds looks unlikely to improve in the foreseeable future. It is, therefore, essential that clinicians are both content and competent in selecting and undertaking the most appropriate treatment for grossly carious primary molars.

The first treatment decision for the young patient with one or more extensively carious primary molars is whether to retain or extract these teeth. Any treatment plan should be based on a thorough history, clinical examination and appropriate investigations. It should also take into account the patient's social, medical and dental status.

A study discusses a technique for managing carious primary molars without major intervention. The Hall Technique (HT) is a method for managing carious primary molars. Decay is sealed under preformed metal crowns without any caries removal, tooth preparation or local anesthesia. The aim of this study was to compare HT clinical/radiographic failure rates with General Dental Practitioners' (GDPs) standard (control) restorations. It was a randomized control trial (132 children, aged 3 to 10 years, GDPs, n = 17) carried out in Scotland. There were 264 study teeth with initial lesions, 42% of which were radiographically > half-way into dentin and 67% of which had class II restorations. Teeth were randomized to HT (intervention) or GDPs usual treatment (control). It was interesting to note that sealing in caries by the HT statistically and clinically, significantly outperformed GDPs' standard restorations in the long term.

Maybe a method we may consider for studying more in depth.


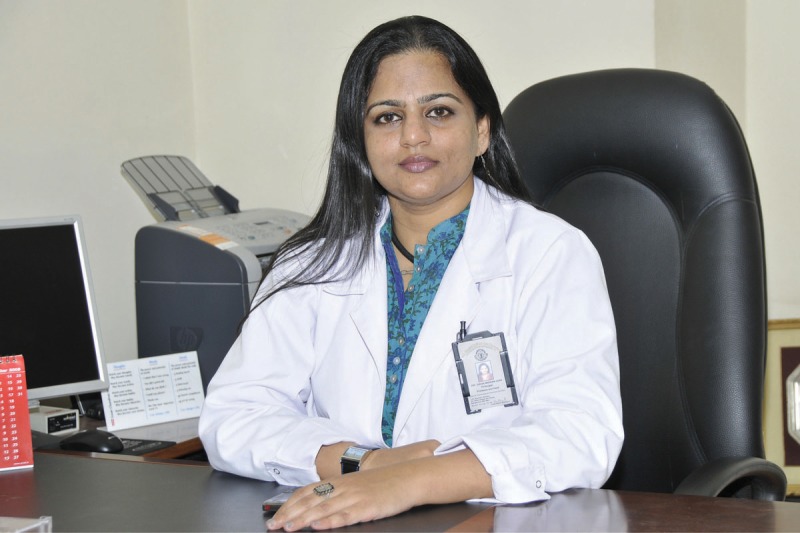
**Usha Mohan Das**
Editor-in-chief

e-mail: ushaamohandas@gmail.com

